# Novel (Hetero)arylalkenyl propargylamine compounds are protective in toxin-induced models of Parkinson’s disease

**DOI:** 10.1186/s13024-015-0067-y

**Published:** 2016-01-13

**Authors:** Mária Baranyi, Pier Francesca Porceddu, Flóra Gölöncsér, Szabina Kulcsár, Lilla Otrokocsi, Ágnes Kittel, Annalisa Pinna, Lucia Frau, Paul B. Huleatt, Mui-Ling Khoo, Christina L. L. Chai, Petra Dunkel, Peter Mátyus, Micaela Morelli, Beáta Sperlágh

**Affiliations:** Laboratory of Molecular Pharmacology, Institute of Experimental Medicine, Hungarian Academy of Sciences, Budapest, Hungary; Department of Biomedical Sciences, Section of Neuropsychopharmacology, University of Cagliari, Cagliari, Italy; János Szentágothai Doctoral School of Neurosciences, Semmelweis University, Budapest, Hungary; National Research Council of Italy, Neuroscience Institute, Cagliari, Italy; Institute of Chemical Engineering and Science, A*STAR, 8 Biomedical Grove, Neuros, Singapore, 138665 Singapore; Department of Pharmacy, National University of Singapore, 18 Science Drive 4, Singapore, 117543 Singapore; Institute of Organic Chemistry, Semmelweis University, Budapest, Hungary

**Keywords:** Propargylamine, Parkinson’s disease, Mitochondrial dysfunction, Mono-aminooxidase B, Neuroprotection, Dopamine

## Abstract

**Background:**

Mitochondrial dysfunction, oxidative stress and their interplay are core pathological features of Parkinson’s disease. In dopaminergic neurons, monoamines and their metabolites provide an additional source of reactive free radicals during their breakdown by monoamine oxidase or auto-oxidation. Moreover, mitochondrial dysfunction and oxidative stress have a supraadditive impact on the pathological, cytoplasmic accumulation of dopamine and its subsequent release. Here we report the effects of a novel series of potent and selective MAO-B inhibitory (hetero)arylalkenylpropargylamine compounds having protective properties against the supraadditive effect of mitochondrial dysfunction and oxidative stress.

**Results:**

The (hetero)arylalkenylpropargylamines were tested in vitro, on acute rat striatal slices, pretreated with the complex I inhibitor rotenone and in vivo, using the 1-methyl-4-phenyl-1,2,3,6-tetrahydropyridine (MPTP) induced acute, subchronic, and chronic experimental models of Parkinson’s disease in mice. The compounds exhibited consistent protective effects against i) in vitro oxidative stress induced pathological dopamine release and the formation of toxic dopamine quinone in the rat striatum and rescued tyrosine hydroxylase positive neurons in the substantia nigra after rotenone treatment; ii) in vivo MPTP-induced striatal dopamine depletion and motor dysfunction in mice using acute and subchronic, delayed application protocols. One compound (SZV558) was also examined and proved to be protective in a chronic mouse model of MPTP plus probenecid (MPTPp) administration, which induces a progressive loss of nigrostriatal dopaminergic neurons.

**Conclusions:**

Simultaneous inhibition of MAO-B and oxidative stress induced pathological dopamine release by the novel propargylamines is protective in animal models and seems a plausible strategy to combat Parkinson’s disease.

**Electronic supplementary material:**

The online version of this article (doi:10.1186/s13024-015-0067-y) contains supplementary material, which is available to authorized users.

## Background

Parkinson’s disease (PD) is a chronic neurodegenerative disorder, characterized by the progressive degeneration of the nigrostriatal dopaminergic pathway. The disease is manifested in various motor symptoms such as bradykinesia, postural instability, resting tremor and muscle rigidity, appearing only at an advanced (70 %) stage of degeneration. Moreover, as the pathology progresses, degeneration extends to other cortical and subcortical non-dopaminergic pathways, responsible for non–motor symptoms such as depression, delusions and cognitive decline.

The current treatment of PD relies on the replacement of dopamine (DA) by its precursor, levo-DOPA (L-DOPA) or dopaminergic agonists [1, 2]. A necessary prerequisite for these approaches, however, is the presence of functioning dopaminergic nerve terminals in the striatum, meaning the disease becomes increasingly refractory to pharmacological intervention through its progression. Moreover, these approaches have many side effects e.g. dyskinesia or motor fluctuations, which cannot easily be tolerated during life-long treatment and could be a starting point for further complications, such as dopamine dysregulation syndrome or impulse control disorder [3]. An alternative approach used in existing therapies is based on the inhibition of enzymes involved in the metabolic degradation of dopamine (monoamine oxidase, MAO, catechol-O-methyltransferase, COMT). This is primarily used as an add-on therapy [4]. Selective monoamine oxidase-B (MAO-B) inhibitors inhibit the enzyme responsible for the intracellular degradation of dopamine, thereby increasing dopamine availability in the nigrostriatal pathway and prolonging the effectiveness of L-DOPA replacement therapy. In addition, certain MAO-B inhibitors, such as selegiline or rasagiline, are thought to be neuroprotective against dopaminergic cell death. Rasagiline, which is a selective and irreversible inhibitor of MAO-B, increases cell survival in cell culture models of PD [5, 6]. This protective effect, however, has not been so convincingly replicated in the clinic [7, 8]. In the recent ADAGIO (Attenuation of Disease Progression with Azilect Given Once-Daily) trial, a delayed start study design was applied to separate symptomatic benefit from disease-modifying effect, however, the results failed to define a clear, dose-dependent disease-modifying action of rasagiline [7]. Despite considerable efforts, at present, there is no single therapeutic agent able to halt or slow down the progression of neurodegeneration in clinical settings [9, 10].

One potential reason for this failure is that the process leading to neuronal death is remarkably complex, involving self-amplifying mechanisms on subcellular, cellular and system levels and drugs that act at a single target are unlikely to have significant influence on the final outcome. [11]. Systemic mitochondrial dysfunction, a subsequent oxidative stress, their interplay and the consequent dysregulation of dopamine pools are core pathological features of PD, which play a determinant role in the development of the relatively selective degeneration of nigrostriatal dopaminergic neurons [12–14]. Systemic mitochondrial dysfunction can be modelled in animal experiments by chronic low dose continuous exposure of rats to the mitochondrial complex I inhibitor rotenone, which reproduces the major anatomical, neurochemical and behavioral hallmarks of Parkinson’s disease in rats [15–17]. More recently, it was confirmed that the same neurochemical alterations could be reproduced by in vitro pretreatment of striatal slices with rotenone, overcoming the weak reproducibility of the in vivo rotenone model [18, 19].

Using rotenone-induced models, we have previously shown that mitochondrial dysfunction and oxidative stress have a supraadditive impact on the pathological, cytoplasmic accumulation of dopamine and its subsequent release in striatal slices from rats [16, 20]. Moreover, in dopaminergic neurons, monoamines and their metabolites provide an additional source of highly reactive free radicals during their breakdown, thereby potentially reinforcing the harmful effects of oxidative stress [20]. Dopamine quinone (DAQ) is generated from released DA in the striatum following in vivo or in vitro rotenone pretreatment, but only under conditions of coincident oxidative stress [16, 19, 20]. Inhibition of mitochondrial complex I by rotenone leads to energy depletion and serves as a source of reactive oxygen species (ROS), causing oxidative damage at multiple target sites (lipid peroxidation, protein and DNA damage, etc.). As a result, DA is redistributed from vesicles and accumulates in the cytosol, from where it is released and provides an additional source of highly reactive free radicals by auto-oxidation or by breakdown by MAO located at the outer surface of mitochondria [21, 22]. Thus, DA is oxidized to the electron-deficient, toxic DAQ, which reacts readily with cellular nucleophiles, such as the sulfhydryl groups of cysteinyl residues of peptides and proteins [23]. Those drugs which simultaneously target mitochondrial dysfunction, oxidative stress and subsequent pathological dopamine release may have disease-modifying potential in addition to symptomatic improvement by the blockade of self-amplifying circuits leading to ROS generation.

Recently, a novel series of (hetero)arylalkenylpropargylamine compounds were synthesized, that demonstrate highly potent MAO-B inhibitory activity with remarkable selectivity over other types of amine oxidase enzymes [24]. In addition, a number of compounds among this series displayed cytoprotective properties against 6-hydroxidopamine (6-OHDA)-induced cell death in PC12 cells. However, culture-based cell survival assays represent oversimplified experimental systems, and they might not reflect the complexity of in vivo systems. Therefore, to extend these findings to in vivo conditions and in order to identify their mechanism of action, in the present study, six compounds were selected among the most promising (hetero)arylalkenylpropargylamines for further evaluation (Table 1). The selection of the compounds was based (1) on their potency and selectivity to inhibit human recombinant MAO-B enzyme and (2) on their protective properties against 6-OHDA induced cell death in PC12 cells reported in our previous study [24]. One compound (SZV558) was selected for more detailed investigation: besides its excellent biological profile including neuroprotective actions in vitro, it proved to be safe in high doses with no hERG and mutagenic activities; moreover it possesses CNS drug-like physicochemical properties with no significant violation of druglikeness scores including Rule-of-Five and Rule-of-Three based on in silico calculation with QikProp (Schrodinger Suite).

**Table 1:** The structural formulae of the test compounds used in the study

Number	Structure
Rasagiline mesylate	
SZV-558	
SZV-2220	
SZV-2358	
SZV2419	
SZV-2435	
SZV-2533	
SZV-557	
SZV-1680	

We have examined the effect of the compounds on oxidative stress induced pathological [^3^H]dopamine release, subsequent [^3^H]DAQ formation and on tyrosine hydroxylase (TH) immunoreactivity in the rotenone pretreated rat striatum. Their effects were also evaluated on dopamine depletion in MPTP- induced acute and subchronic mouse models of Parkinson’s disease. However, a number of studies have indicated that the regimen of MPTP administration is fundamental to reproduce parkinsonian symptoms and that its chronic administration induces a gradual and persistent degeneration of nigrostriatal neurons associated with motor deficit [25, 26]. Accordingly, chronic exposure to low doses of MPTP over several weeks, in combination with the clearance inhibitor probenecid, has been shown to reproduce several aspects of the human disease and to be a most suitable model for studying drugs with neuroprotective potential [27–29].

On this basis, we have also evaluated the efficacy of SZV558 compared with rasagiline in a chronic mouse model of MPTP plus probenecid (MPTPp) in which a progressive development of parkinsonian symptoms, neurodegeneration, and neuroinflammation has been reported [29]. Similarly to PD, this model has shown that a progressive neurodegeneration is associated with a gradual development of symptoms, from olfactory dysfunction to motor impairment, providing a useful tool to test neuroprotective therapies aimed at modifying disease progression [30].

## Results

### Effect of the compounds on oxidative stress-induced pathological [^3^H]dopamine release in the rotenone pretreated rat striatum

**Fig. 1:**
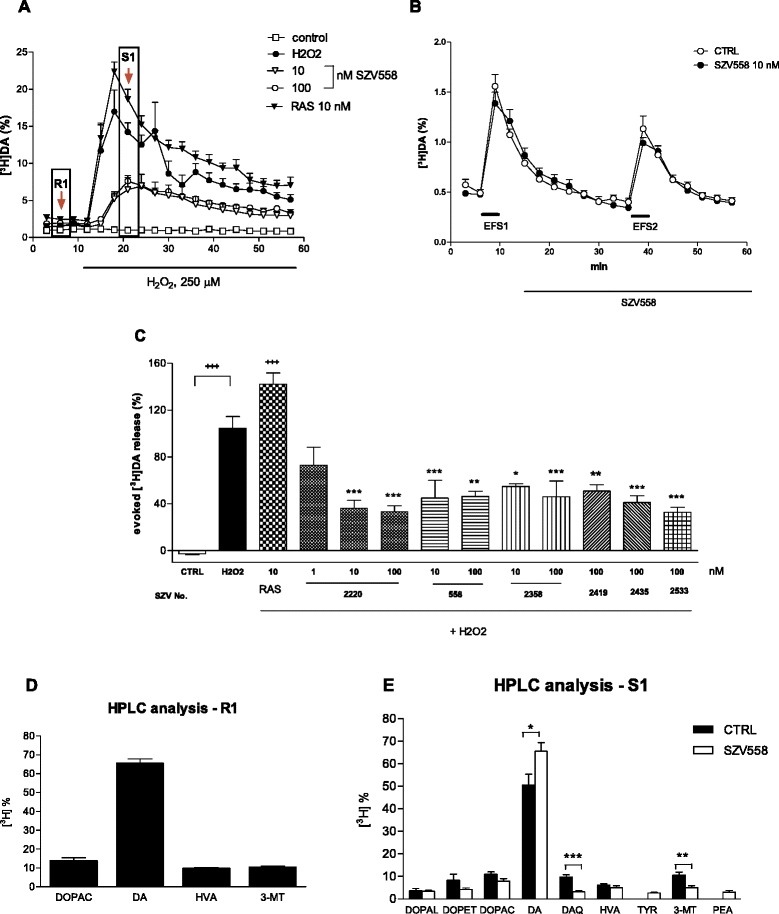
Effect of test compounds on oxidative stress induced pathological tritium release after rotenone treatment (**a**, **c**); on electrical field stimulation (EFS) evoked [^3^H]DA release (**b**) and on the tritium composition in the effluent (**d**, **e**) in rat striatal slices. **a**, **b**. Oxidative stress was mimicked by the perfusion of H_2_O_2_ (250 μM), as indicated by the horizontal bar on **a**. Control slices were perfused identically with Krebs’ solution, but without H_2_O_2_. Test compounds or their vehicle (CTRL) were administered 18 min before the perfusion with H_2_O_2_ and onwards. **b**. SZV558 or its vehicle (CTRL) was administered 18 min before EFS2 and onwards. The release of [^3^H]DA and its tritiated metabolites is expressed as fractional release (%). *N* = 4-8 independent experiment/group. **c** When the effects of drugs were compared on H_2_O_2_ evoked tritium release, the net release evoked by H_2_O_2_ calculated by the area-under-the-curve method was taken into account. *N* = 4-8 independent experiment/group. Symbols represent significant changes from control slices (^+++^ *P* < 0.001), and H_2_O_2_ treated slices (*P < 0.05, **P < 0.01, ***P < 0.001), respectively. Statistical analysis: one-way ANOVA followed by the Tukey test. **d**, **e** The samples labelled by arrows (R1, S1) on **a** were analyzed by HPLC to identify the composition of the tritium label. The composition of the tritium efflux is expressed as percentage of the total tritium efflux. *N* = 6-8

At first, we have evaluated the six selected compounds in vitro and addressed the question, whether they counteract the supraadditive effect of mitochondrial dysfunction and oxidative stress on subsequent pathological [^3^H]dopamine efflux from superfused rat striatal slices. After rotenone preincubation (10 μM, 60 min) and 60 min preperfusion the basal tritium efflux was 1.014 ± 0.08 % (Fig. 1a, *n* = 4) and remained relatively constant throughout the sample collection period. Whereas a switch to an identical medium did not change [^3^H]DA efflux (control), oxidative stress, simulated by H_2_O_2_ (250 μM) perfusion elicited a remarkable increase in the release of tritium (Fig. 1a). In line with literature data [19], the selective MAO-B inhibitor, rasagiline (10 nM), did not decrease oxidative stress-induced pathological tritium release (Fig. 1a and c). In contrast, novel propargylamine compounds (1 nM-100 nM) had a significant inhibitory effect on the oxidative stress induced pathological dopamine release in rat striatum slices in nanomolar concentrations (Fig. 1a, c). In another set of experiments, slices were subjected to electrical field stimulation (EFS1, EFS2, 25 V, 1 msec, 2 Hz, 240 shocks) twice to model physiological neuronal activity, and SZV558 was administered 18 min before EFS2 and onwards (Fig. 1b). EFS elicited a rapid and reproducible efflux of [^3^H]DA. However, SZV558 did not affect [^3^H]DA release evoked by EFS, illustrating its differential effect on pathological and physiological DA release (Fig. 1b).

To identify the formation of physiological and toxic dopamine metabolites in the effluents, selected samples collected under basal conditions and during the peak of the effect of H_2_O_2_ were subjected to further HPLC analyses. Separation of tritium label in the effluent revealed that in samples collected under basal conditions, the tritium label contained [^3^H]DA and its metabolites [^3^H]DOPAC, [^3^H]HVA, and [^3^H]3-MT (Fig. 1d). HPLC analyses of the samples, which were collected after the perfusion with H_2_O_2_, showed that [^3^H]DAQ and other metabolites of dopamine, such as [^3^H]DOPAL and [^3^H]DOPET also appeared in the effluent with a simultaneous decrease of [^3^H]DA under these conditions, the proportion of which decreased in these samples (50.66 ± 4.69 %, *n* = 6, and Fig. 1e), when compared to the effluent collected under resting conditions (65.64 ± 2.25 %, *n* = 6). SZV558 (100 nM) significantly reduced the formation of DAQ and elevated the proportion of dopamine in the effluent (Fig. 1e), indicating its protective effect against the harmful interaction between mitochondrial dysfunction and oxidative stress.

**Fig. 2:**
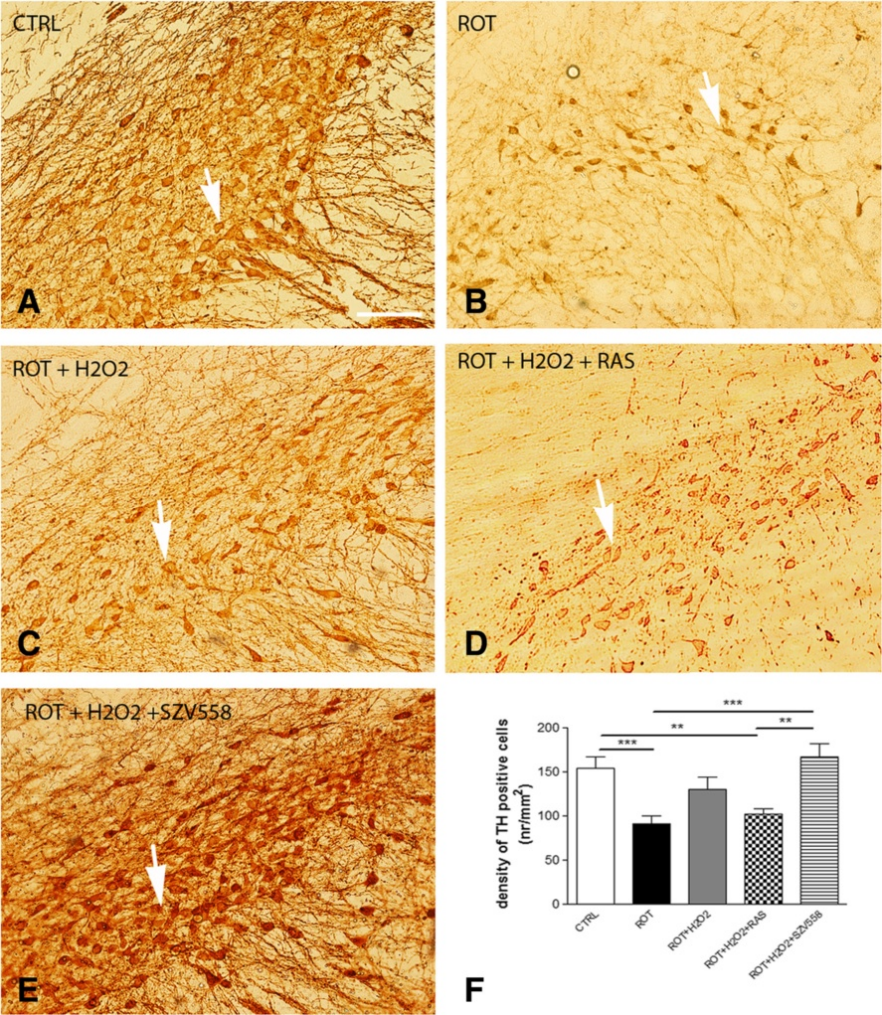
Tyrosine hydroxylase (TH) immunostaining in rat SNc after in vitro rotenone (ROT) (**b**) rotenone + H_2_O_2_ (**c**), rotenone + H_2_O_2_ + rasagiline (RAS) (**d**) and rotenone + H_2_O_2_ + SZV558 treatment (**e**). The caudal part of the brain containing the SNc was prepared from rats and then the experiment was continued according to the protocol described in the Methods. After the experiment, 40 μm coronal vibratomic midbrain sections were prepared and processed for TH immunostaining. **a** Immuno-DAB staining for TH on control tissue sections. Immunoreactivity is seen in the cell body and processes of dopaminergic and noradrenergic neurons. **b** Due to rotenone treatment both the number of immunolabelled neurons and the intensity of staining is substantially decreased. The cell bodies of stained neurons are smaller than in the control sample. **c** Weak immunostaining shows some TH-expressing neurons on the rotenone + H_2_O_2_treated section. **d** TH immunoreactivity in SNc after rotenone + H_2_O_2_ + rasagiline treatment. **e** Intensity of DAB immunostaining for TH on the SN area looks similar to the control tissue after rotenone + H_2_O_2_ + SZV558 treatment of the tissue. Arrows show cell body of dopaminergic/noradrenergic neurons. Original magnification: 20X, bar: 100 μm **f**. Quantification of the TH immunostaining. Digitalization was performed by means of a Pannoramic P250 scanner (3DHISTECH, Budapest, Hungary) using 9 optical layer with a resolution of 0.11 μm/pixel. Pictures were taken at five representative areas in each of samples at 40× magnifications. The stained cells were counted manually, marked with a marker counter function of the Pannoramic Viewer 1.15.4 digital slide viewer software application provided by 3DHISTECH, Budapest, Hungary and expressed as number/mm^2^ (nr/mm^2^). Statistical analysis: one-way ANOVA followed by the Tukey test. **P < 0.01, ***P < 0.001, as indicated by the horizontal bars. *N* = 4–5 /group

As a readout of neuroprotective effect, TH immunostaining was also performed on tissue blocks containing the *substantia nigra pars compacta* (SNc) after identical treatments (Fig. 2). In line with expectation, substantially less dopaminergic neurons stained for TH after rotenone (ROT) treatment and a decreased volume of cell body was also observable (*P* < 0.001 vs. CTRL, Fig. 2b, f), compared to control (CTRL) sections. There was no further decrease in intensity of TH immunostaining or difference in the shape of stained cell bodies in samples treated with rotenone + H_2_O_2_ (Fig. 2c). Treatment with rasagiline (100 nM) did not significantly change the effect of rotenone + H_2_O_2_ on TH immunoreactivity (*P* > 0.05 vs ROT+ H_2_O_2_, *P* < 0.01 vs CTRL, Fig. 2d, f). In contrast, in samples treated with SZV558, the number of TH positive neurons was similar to the control sample, demonstrating the rescuing effect of SZV558 on dopaminergic neurons against the toxic compound rotenone (*P* < 0.001 vs. ROT, *P* > 0.05 vs. CTRL and rotenone + H_2_O_2_, *P* < 0.01 vs. rasagiline, Fig. 2e, f).

### The protective effect of the compounds in acute MPTP-induced striatal dopamine depletion and motor deficit in mice

Next, we evaluated the effect of test compounds on the level of endogenous DA and its metabolites by HPLC analyses in in vivo murine PD models. The effect of two compounds (SZV558 and SZV2220), displaying high potency at both rodent and human MAO-B [24] were also examined on MPTP/MPP+ levels and on motor function. Whilst all the other compounds were used as a single i.p. pretreatment in 10 mg/kg dose prior to MPTP regimen, in case of SZV558, the most promising compound according to previous in vitro data, dose-dependence, *per os* route of administration and duration of action were also tested.

**Fig. 3:**
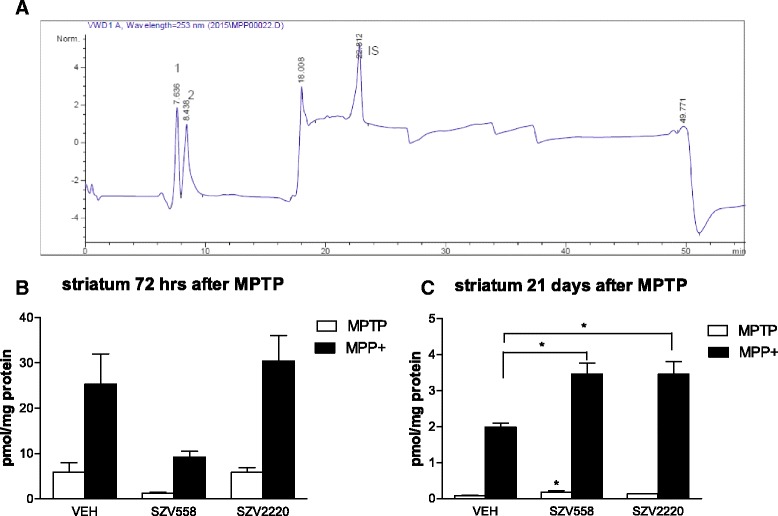
Effect of SZV compounds on the level of MPTP and MPP^+^ after an acute (**b**) and subchronic (**c**) MPTP treatment protocol. **a** Representative chromatogram generated by UV detection at 253 nm, showing MPTP (1) and MPP^+^ (2). Theophylline was used as internal standard (IS). **b** Animals were pre-treated with test compounds or their vehicle (VEH) i.p. 18 h before the first dose of MPTP; then treated with MPTP 4×20 mg/kg i.p. 2 h apart. MPTP and MPP^+^ levels were analyzed in striatum samples obtained 72 h after the last dose of MPTP **c**. Mice were treated with MPTP (30 mg/kg i.p. daily) for 5 days and then, for 21 days with the test compounds or their vehicle (VEH) i.p. and sacrificed 1 h after the last treatment. MPTP and MPP^+^ were analyzed by HPLC and expressed as pmol/mg protein. Note that the y scale in **b** and **c** is different. *N* = 6-8/group. Symbols represent significant changes from vehicle treated rats (*P < 0.05). Statistical analysis: one-way ANOVA followed by the Tukey test

To study the effect of the compounds under in vivo conditions, at first, we took advantage a widely used acute MPTP protocol (4x20 mg/kg i.p. 2 h apart), and test compounds were administered 18 h before the start of MPTP treatment. Animals were sacrificed 72 h after the last MPTP dose and the amount of MPTP and MPP^+^ in striatal samples were analyzed by HPLC-UV (Fig. 3a-b). The majority of MPTP has already been converted to MPP^+^ at this time point, (Fig. 3b, 25.29 ± 6.65 pmol/mg protein, *n* = 8), although MPTP was still clearly detectable (5.87 ± 2.19 pmol/mg protein, *n* = 8). Although there was a tendency of reduction of both MPTP and MPP^+^ in case of SZV558, no significant difference were detected in their amount in mice that have been pretreated with SZV2220 or SZV558 18 h before the start of MPTP treatment when compared to vehicle treated animals (Fig. 3b).

**Fig. 4:**
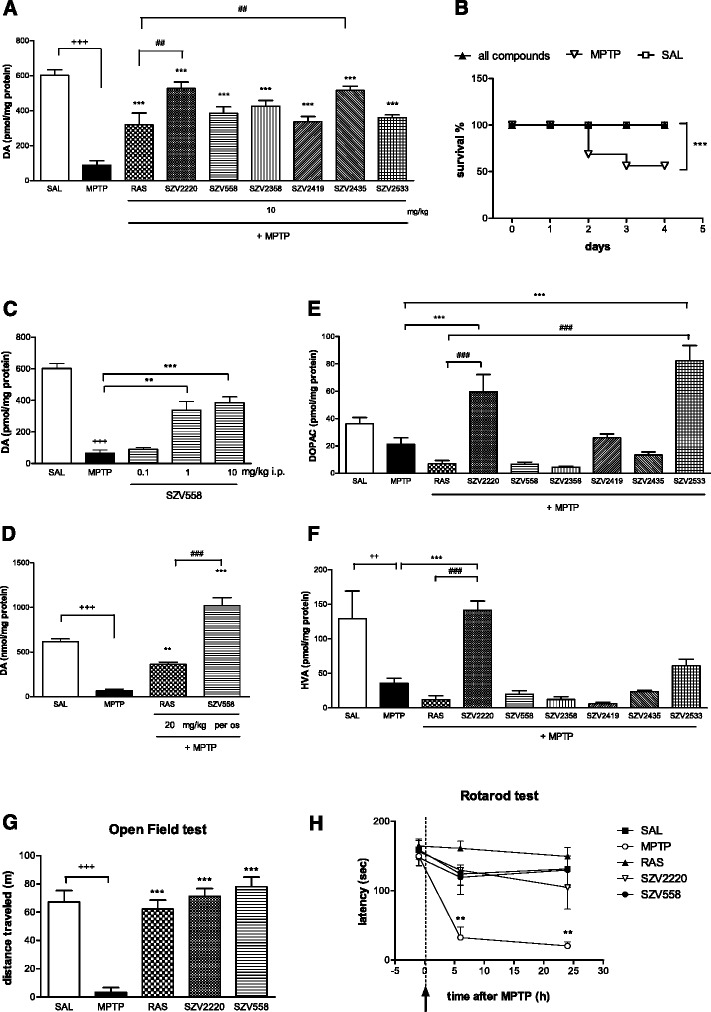
Effect of SZV compounds on endogenous dopamine content in the striatum (**a**, **c**, **d**); on the survival of mice (**b**); on endogenous dopamine metabolite (DOPAC, HVA) levels (**e**, **f)**; on the basal locomotor activity (**g**) and on motor coordination (**h**) after in vivo MPTP treatment. **a** Effect of test compounds and rasagiline (10 mg/kg i.p. each) on endogenous dopamine level in the striatum; **b** on the survival of the animals. Survival is expressed as percentage of the initial number of animals. **c** Dose-dependent effect of SZV558 (0.1-10 mg/kg i.p.) on endogenous dopamine content in the striatum. **d** Effect of SZV558 and rasagiline (20 mg/kg *per os* each) on endogenous dopamine content in the striatum. **a**, **c**, **d** Dopamine content is expressed as pmol/mg protein. **e**, **f** Effect of SZV compounds (10 mg/kg i.p.) on endogenous dopamine metabolite (DOPAC, HVA) levels. Metabolite levels are expressed as pmol/mg protein. **g** Effect of SZV compounds (10 mg/kg i.p.) on the basal locomotor activity, measured in the open field test. Locomotion is expressed as the distance traveled during the whole 30 min test period. **a**-**g** Symbols represent significant changes from saline treated (^++^ *P* < 0.01, ^+++^ *P* < 0.001), MPTP treated (**P < 0.01, ***P < 0.001) and rasagiline (10 mg/kg) treated (^##^ *P* < 0.05, ^###^ *P* < 0.001) animals, respectively. Statistical analysis: one-way ANOVA followed by the Tukey test, except **b**: log rank test. Number of independent experiments: 5-12/group. **h** Effect of SZV compounds (10 mg/kg i.p.) on the motor performance measured on the rotarod test. The time elapsed until the falling of the mice was expressed in sec. Symbols represent significant changes from respective pretreatment values (**P < 0.01). Statistical analysis: one-way ANOVA followed by the Dunnett test

To assess whether the compounds are able to protect against the effects of MPTP and preserve the functionality of nigrostriatal dopaminergic pathways, endogenous dopamine contents were analyzed in the striatum by HPLC. MPTP treatment on mice pretreated with saline, elicited a loss of approximately 85 % of endogenous dopamine content in the striatum, accompanied by a decrease in the survival of the animals (Fig. 4a, b). In contrast, mice pretreated with test compounds, (10 mg/kg i.p. each) were in overall good health following MPTP treatment, and none of the drugs produced any obvious untoward behavioral alteration at the dose used. Moreover, the test compounds significantly protected against the effect of in vivo MPTP treatment on the depletion of striatal dopamine content i.e. against the hallmark feature of PD (Fig. 4a) and also completely restored the survival of animals during the course of the experiment (Fig. 4b). The effect of SZV558 was dose-dependent and displayed a tendency of saturation at the 10 mg/kg dose (Fig. 4c). SZV558 was also active using *per os* application (20 mg/kg, Fig. 4d) and completely restored the dopamine content of the striatum above the level of mice treated only with saline but not MPTP (Fig. 4d). At this dose, SZV558 was significantly more effective than rasagiline (*P* < 0.001).

To get a further insight on the action of test compounds, their effects were also examined on the level of dopamine metabolites in the striatum. In line with literature data [31], MPTP treatment tended to reduce the level of dopamine metabolites DOPAC and HVA (Fig. 4e and f), although this was only statistically significant in case of HVA (Fig. 4f). Rasagiline (10 mg/kg i.p.), did not restore the level of metabolites in the presence of MPTP, but further decreased the level of DOPAC and HVA. In contrast, SZV2220 and SZV2533 increased DOPAC levels, (Fig. 4e), and HVA levels were also restored by SZV2220 (Fig. 4f).

To examine motor function, two widely used tests, i.e. the open field and the rotarod test were used. Deteriorated motor function in MPTP-treated animals was restored by SZV2220 and SZV558 (Fig. 4g and h). 2 h following the final MPTP treatment, the basal locomotor activity of mice was minimal, i.e. they displayed akinesia (Fig. 4g). In contrast, mice pretreated with SZV558 and SZV2220 (10 mg/kg i.p. each) showed a profound increase in basal locomotor activity, even at this early time point (Fig. 4g).

In the rotarod test, mice treated with MPTP displayed a time-dependent decrease in motor performance, compared to values measured before MPTP treatment (Fig. 4h). This progressive decline in performance was almost completely prevented by both SZV558 and SZV2220 and was maintained up to 24 h after drug injection (Fig. 4h). Within the time frame examined, the effects of test compounds on motor function were comparable to that of rasagiline.

In order to estimate their duration of action, SZV558 and rasagiline were applied in a single dose (10 mg/kg i.p.) 2 h, or 42 h before the first dose of MPTP in another set of experiment (Additional file 1: Table S1). A slight protective effect against dopamine depletion elicited by MPTP was already detected 2 h after injection of SZV558, although this effect was not statistically significant. The maximum of the effect of SZV558 was obtained 18 h after application. The effect of SZV558 then declined and a tendency of protection was still detected 42 h after SZV558 administration. These data indicate the irreversible inhibition of the MAO-B enzyme by SZV558; rasagiline, which is also an irreversible inhibitor of the MAO-B enzyme, had a similar duration of action (Additional file 1: Table S1). Among SZV557 and SZV1680, the two major potential metabolites of SZV558, SZV557 displayed partial activity at the MAO-B enzyme, whereas SZV1680 proved to be much less active at both MAO isoforms [24]. In the in vivo MPTP model, SZV557 was partly active, whilst SZV1680 remained completely inactive in the restoration of endogenous dopamine content of the striatum, when added 18 h before the first MPTP injection. Regarding survival, SZV557 was fully active, whilst SZV1680 only marginally affected the survival of the animals. This finding indicates that the propargyl-moiety of the compounds is instrumental for their antiparkinsonian effect.

### The protective effect of SZV558 in subchronic MPTP-induced striatal dopamine depletion

Next, a more complex, delayed rodent MPTP protocol [32, 33] was utilized to examine whether the protective effect of the compounds (1) is entirely due to their MAO-B inhibitory action and (2) is prolonged for a long time and is extended to apoptotic neuronal death characteristic of human PD. Using the protocol described by Tatton and Kish [32, 33], mice were treated daily with MPTP (30 mg/kg i.p) for 5 consecutive days and test compounds or their vehicle were delivered for 21 days only after then, when the majority of MPTP is expected to be converted to the toxic MPP^+^ [33, 34]. Using this protocol, MPTP and MPP^+^ detected in these samples was less than 10 % detected 72 h after the last MPTP dose in the acute model, although still present, with a low MPTP/MPP^+^ ratio (<5 %, MPTP: 0.08 ± 0.01 pmol/ mg protein, *n* = 8, MPP^+^: 1.99 ± 0.10 pmol/mg protein, *n* = 8) (Fig. 3c). Once again, test compounds, i.e., SZV558 and SZV2220 did not decrease the level of MPP^+^ detected in the samples (Fig. 3c), MPP^+^ levels were significantly higher in samples collected from mice treated with the test compounds than in mice treated with vehicle.

**Fig. 5:**
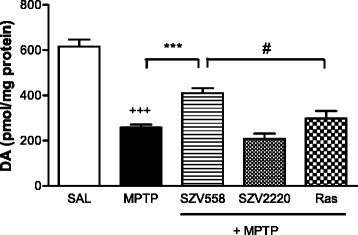
The effects of SZV558, SZV2220 and rasagiline (10 mg/kg i.p for 21 days each) on MPTP treated mice striatum using a subchronic (Tatton-Kish) model of PD. The MPTP group received vehicle treatment for the same period after the MPTP treatment. Dopamine content is expressed as pmol/mg protein. Symbols represent significant changes from saline treated (^+++^ *P* < 0.001), MPTP treated (***P < 0.001) and rasagiline treated animals, (^#^ *P* < 0.05), respectively. Statistical analysis: one-way ANOVA followed by the Tukey test. Number of independent experiments: 8-12/group

Application of MPTP treatment using this method resulted in a̴ 60 % reduction of endogenous dopamine content in the striatum, when compared to saline treatment (Fig. 5). SZV558, but not the reference compound rasagiline (10 mg/kg/day i.p. each, for 21 days), significantly restored the endogenous dopamine content (Fig. 5).

### Effect of SZV558 in a chronic MPTPp induced mouse model of PD

Since PD is a chronic neurodegenerative disease, SZV558 was compared with rasagiline in a chronic mouse model of MPTP plus probenecid (MPTPp) characterized by the progressive development of parkinsonian symptoms, neurodegeneration, and neuroinflammation [29]. To investigate whether the impairments of motor performance and olfactory dysfunction induced by MPTPp were reverted by SZV558, we evaluated motor performance and olfactory function with the beam-walking test, the inverted grid test, the motility test, and the pellet retrieval olfactory test. Moreover, the neuroprotective efficacy of SZV558 on dopaminergic neuron degeneration was assessed in the striatum and the SN pars compacta (SNc) by TH immunohistochemistry.

#### Changes in spontaneous motor activity: motility test

To evaluate the modification of the spontaneous motor activity induced by the treatment, the total activity and the ambulatory activity were evaluated through the motility test before the MPTPp treatment and 1 day after the last MPTPp administration. We observed no significant changes for total activity (Table 2) and ambulatory activity (Table 2) after MPTPp administration. Moreover, in animals treated with MPTPp compared with vehicle and MPTPp plus SZV558 or rasagiline groups, the one-way ANOVA did not indicate a significant effect of treatment in the ambulatory activity.

**Table 2:** Motility test pre- and post-chronic MPTPp treatment

TOTAL ACTIVITY				
	Pre-treatment		Post-treatment	
	Mean	SEM	Mean	SEM
Vehicle	11503.75	983.49	8737.50	1651.77
MPTPp	5803.80	1162.18	6587	771.63
MPTPp + SZV558	9063.14	1055.95	7904.85	982.36
MPTPp + Rasagiline	9941.57	1055.57	8867.85	1214.24
LOCOMOTOR ACTIVITY				
Vehicle	8727	983.49	3690.25	1651.77
MPTPp	4004.60	916.64	2773.40	343.84
MPTPp + SZV558	6929.85	791.63	3415.86	463.68
MPTPp + Rasagiline	7497	928.82	3938	639.83

Motor activity was measured 2 days before the first MPTPp administration and 1 day after the last MPTPp administration. Values are expressed as mean ± SEM

Data were statistically compared with one-way ANOVA, followed by the Newman–Keuls post hoc test

Number of independent experiments: 4–7/group

#### Effect of motor impairment induced by MPTPp: beam-walking test

**Fig. 6:**
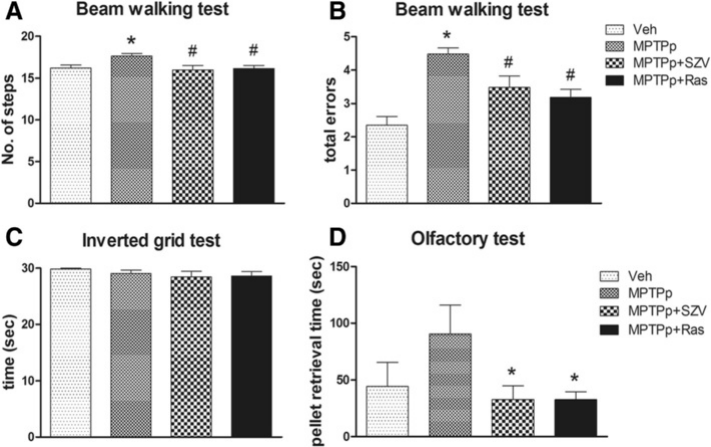
The effect of SZV558 on the behavior in the chronic MPTPp induced PD in mice. Adult mice were treated with vehicle plus probenecid, or MPTP (25 mg/kg i.p.) plus probenecid (100 mg/kg i.p.), administered 30 min before each MPTPp, twice a week for 5 weeks, alone, or in the presence of the SZV558 (1 mg/kg i.p.) or rasagiline (1 mg/kg i.p.) administered 18 h before each MPTPp administration. **a**-**b** Beam-walking test. To evaluate the number of steps (**a**) and the total errors (**b**), the mice were videotaped for a total of five trials, 2 days after the 10^th^ MPTPp administration. The number of steps and the number of errors were calculated across all five trials and averaged for each group. * *P* < 0.05 compared with vehicle, # *P* < 0.05 compared with MPTPp. **c** Inverted grid test. To assess skilled forepaw use, the latency time of falling down was measured 2 days after the 10^th^ MPTPp administration. **d** Olfactory test. To evaluate the olfactory deficit, the retrieval time of a buried smelling pellet was measured 3 days after the 10^th^ MPTPp administration. * *P* < 0.05 compared with MPTPp. Values are expressed as mean ± SEM. Data were statistically compared with one-way ANOVA, followed by the Newman-Keuls post hoc test. N: 4–7/group

The number of steps and errors were recorded for each animal 2 days after the 10^th^ MPTPp administration, in order to evaluate, respectively, the motor performance and coordination. Despite, the analysis of the time to traverse the beam did not show any difference between the four groups (data not shown). The analysis of the number of steps showed an increase in the MPTPp group. The post hoc test (Newman-Keuls) showed that treatment with SZV558 or rasagiline prevented the impairment induced by MPTPp (Fig. 6a).

Analysis of the total errors showed that MPTPp administration induced a significant increase in total errors compared with vehicle, demonstrating motor function impairment (Fig. 6b). Motor function improvement, demonstrated by the decrease in total errors, was observed in the groups treated with SZV558 or rasagiline compared with the MPTPp group, as indicated in the post hoc test.

#### Effect of grasp-strength evaluation: inverted grid test

The inverted grid test was used to assess forepaw use, especially related to distal musculature and digit manipulations. This test was done 2 days after the end of treatment. No significant changes with either MPTPp or rasagiline were observed, indicating that the mice did not have any distal musculature deficit (Fig. 6c).

#### Effect of SZV558 on olfactory deficit induced by MPTPp

To reveal any effect of the compounds on olfactory deficit, elicited by MPTPp, mice were evaluated for olfactory function 3 days after the end of treatment, through the retrieval time of a buried smelling pellet.

The results of olfactory test showed an increase of retrieval time of a buried smelling pellet, demonstrating an olfactory deficit, in the MPTPp group. In mice treated with MPTPp plus SZV558 or rasagiline, a decrease in retrieval time was observed compared with the MPTPp group (Fig. 6d).

#### TH immunohistochemistry and Nissl Staining in the SNc and striatum

**Fig. 7:**
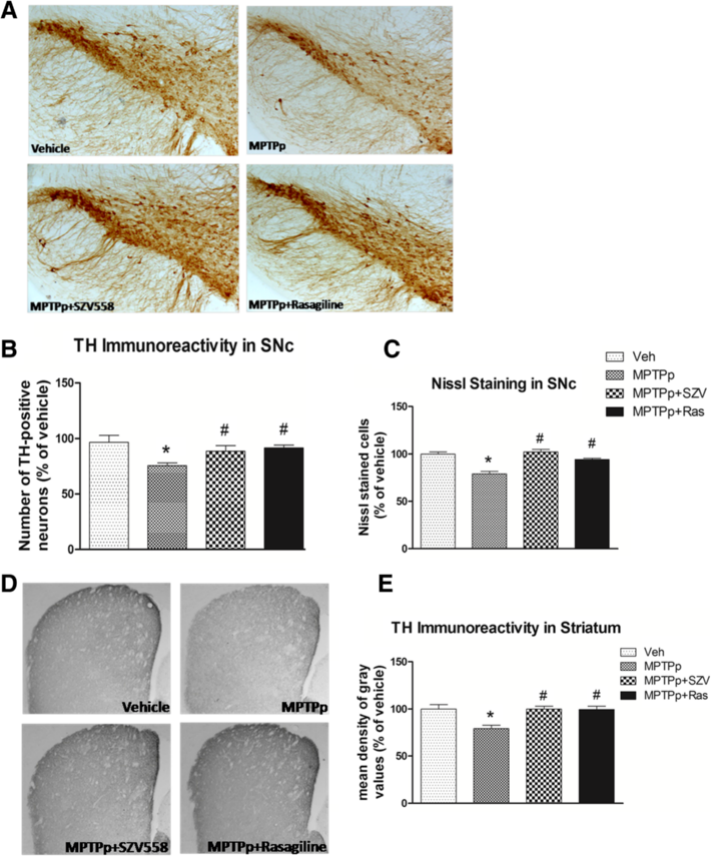
Immunoreactivity for TH in the SNc and in the striatum. Mice were treated with MPTPp alone or in the presence of SZV558 or rasagiline, and sacrificed 3 days after the 10^th^ MPTPp administration. Coronal sections were processed for TH immunostaining as described in the Materials and Methods. Representative coronal sections of SNc immunostained for TH show the TH positive neuronal reduction induced by MPTP treatment (**a**). The graphs show the number of TH immunoreactive cells (**b**) and of Nissl-stained cells (**c**) in the SNc, expressed as a percentage with respect to vehicle-treated mice. SZV558 and rasagiline prevented the loss of TH-positive neurons in the SNc, as compared with the animals treated with MPTPp. Representative coronal sections of striatum immunostained for TH at 5× magnification (**d**). The graph shows the mean density of gray value of TH expressed as a percentage with respect to vehicle-treated mice (**e**). The TH density reduction confirms the neurodegeneration induced by MPTP. The two compounds prevent the loss of TH positive fiber induced by MPTPp. Values are expressed as mean ± SEM. * *P* < 0.05 compared with vehicle. # *P* < 0.05 compared with MPTPp. Data were statistically compared with one-way ANOVA, followed by Tukey’s post hoc test. N: 4–7/group

Next, to evaluate the effect of test compounds on dopaminergic cell death, TH immunohistochemistry and Nissl Staining were performed in the SNc and striatum. MPTPp induced a significant loss of TH-positive cells in the SNc, as measured by TH immunoreactivity (Fig. 7a). Analysis of Nissl-positive nigral neurons showed the neuronal death of MPTPp treated mice compared to vehicle, confirming the dopaminergic neuron degeneration induced by MPTPp. The post hoc analysis (Tukey’s test) of TH immunoreactivity showed that the combined treatment with MPTPp plus SZV558 or rasagiline prevented the loss of dopaminergic neurons in the SNc, observed in the animals treated with MPTPp alone (Fig. 7b). The cresyl violet staining (Fig. 7c) confirmed the loss and the recovery in number of neurons in the SNc in MPTPp and MPTPp plus SZV558 or rasagiline treated mice (Fig. 7c).

The loss of TH-positive fibers in the striatum of mice treated with MPTPp confirmed dopaminergic neuron degeneration (Fig. 7d). Both SZV558 and rasagiline prevented neurodegeneration in the striatum compared with the MPTPp group, as shown by the post hoc analysis (Tukey’s test) of TH-positive fibers in the striatum.

## Discussion

Parkinson’s disease (PD) is a chronic neurodegenerative disease, characterized by motor and non-motor symptoms, which progressively diminish quality of life and the ability to work, and lead to permanent disability. Although these symptoms can be improved using currently available dopamine replacement strategies, treatments that provide neuroprotection and/or disease-modifying effects remain an urgent unmet clinical need [9].

Among in vivo rodent animal models of PD, toxin-induced models (e.g. MPTP, rotenone, 6-OHDA) elicit substantial and reproducible degeneration of catecholaminergic cells and are therefore widely used for testing protective therapeutic interventions [35, 36]. In the present study, the effects of six representatives of a novel series of propargylamine compounds and, as a reference compound, rasagiline were evaluated in several different toxin-induced PD models. In addition, one compound (SZV558) was investigated in more details to identify its impact on the formation of toxic [^3^H]DAQ and on the survival of substantia nigra dopaminergic neurons after in vitro rotenone pretreatment, and to be the subject of a more detailed pharmacological characterization in the in vivo acute MPTP model and in a chronic MPTP model. In our previous study SZV558 inhibited both rat and human MAO-B with high potency (IC_50rat_: 50 nM, IC_50human_: 60 nM) and reasonable selectivity (human MAOA/B selectivity ratio: 58), and elicited a 66 % increase in the survival of PC12 cells treated with 6-OHDA [24].

In cases of test compounds, a consistent protective effect was detected in all models, which was quantitatively and in some cases, qualitatively superior to rasagiline. Moreover, the protective effect was manifested in various forms of indicators from dopamine depletion to animal behavior, without the appearance of any detectable detrimental side effects.

**Fig. 8:**
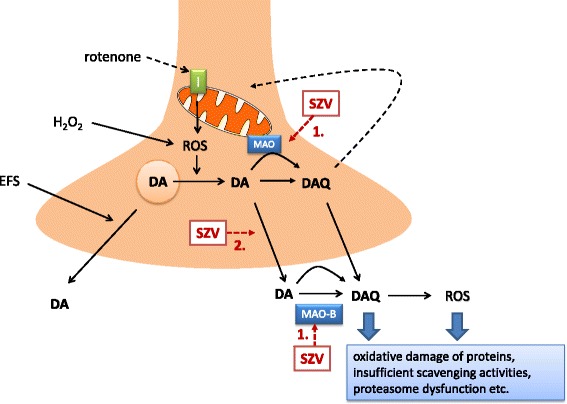
Schematic drawing illustrating the potential mechanism of action of novel (hetero)arylalkenylpropargylamines (SZV) in the striatum. Mitochondrial deficit, caused by the mitochondrial complex I inhibitor rotenone leads to reactive oxygen species (ROS) generation. Coincident or subsequent oxidative stress, mimicked in our experiments by H_2_O_2_ leads to redistribution of DA from synaptic vesicles to cytoplasm, where it is converted to toxic dopamine quinone (DAQ). DA is then released to the extracellular space. DAQ targets the cysteinyl residues of peptides and proteins and compromise cellular function at multiple target sites and aggravates mitochondrial deficit. SZV compounds 1. inhibit MAO-B enzyme, and thereby increase the biological availability of dopamine; 2. in addition, they prevent oxidative stress induced pathological DA efflux and its further formation to toxic DAQ, independent from the inhibition of MAO-B, but do not have direct antioxidant property and do not influence physiological neuronal activity induced DA release, mimicked by electrical field stimulation (EFS)

As a potential mechanism of action, all compounds inhibited oxidative stress-induced pathological dopamine release in the rotenone treated rat striatum slices (Fig. 8). In our previous studies we established that mitochondrial dysfunction and oxidative stress have a supraadditive impact on the pathological, cytoplasmic accumulation of dopamine and its subsequent release [16, 20]. Moreover, in dopaminergic neurons, monoamines and their metabolites provide an additional source of highly reactive free radicals during their breakdown by MAO or auto-oxidation, thereby potentially reinforcing the harmful effects of oxidative stress [37]. In this study, we have confirmed these results and revealed that test compounds inhibited this pathological [^3^H]DA efflux, without affecting [^3^H]DA release induced by the low frequency electrical stimulation modelling physiological neuronal activity. Test compounds were effective in a low concentration, in contrast to rasagiline, which remained ineffective in this respect. Because both rasagiline and the test compounds potently inhibit MAO-B, these findings indicate that the putatively protective action of the test compounds is partly independent from inhibition of MAO-B.

In case of SZV558, we have also analysed the tritium composition under resting conditions and during the peak of the effect of H_2_O_2_. Whilst in the effluent collected under basal conditions [^3^H]DA is partly converted to major metabolites of dopamine i.e. [^3^H]DOPAC, [^3^H]3-MT and [^3^H]HVA, in the effluent collected under the action of H_2_O_2_, other metabolites, such as [^3^H]DOPET and [^3^H]DOPAL also appeared. Moreover, the toxic [^3^H]DAQ is also formed under these conditions but only in the case of precedent mitochondrial dysfunction, as we have shown previously [16, 20]. A peculiar action of SZV558 was the inhibition of the formation of [^3^H]DAQ, with the preservation of a higher proportion of [^3^H]DA in the effluent. The harmful effect of DAQ is well documented as an effector molecule of dopaminergic neuron-specific cytotoxicity [38, 39]. In general, the targets of catechol quinones are cysteine residues of peptides or proteins, and quinones conjugate with their sulfhydryl groups resulting in the formation of 5-cysteinyl-catechols. This covalent modification can irreversibly compromise protein function and is therefore cytotoxic [40]. DAQ and its downstream metabolites reduce mitochondrial function and open the mitochondrial permeability transition pore [41–43]. DAQ also inhibits the function of endogenous ROS scavenging enzymes such as glutathione peroxidase [44] and superoxide dismutase [45]. In addition DAQ inhibits proteasomal activity [46] and inactivates functional proteins of dopaminergic neurons such as TH [47, 48], dopamine transporter [49] and α-synuclein [50]. SZV558 was able to decrease the formation of [^3^H]DAQ, with the preservation of more [^3^H]DA in the effluent, therefore one can assume that it specifically interacts with factors increasing the vulnerability of dopaminergic neurons.

As a histological marker of ongoing neurodegeneration in response to in vitro rotenone treatment, TH immunostaining was examined in the SNc. Our findings revealed that SZV558 not only alleviated DAQ formation in the striatum but was also able to rescue SNc dopaminergic neurons from the toxic effect of rotenone as shown by the restoration of TH immunostaining in tissue samples following SZV558 treatment after rotenone preincubation. Interestingly, H_2_O_2_ treatment did not exacerbate the neurotoxic effect of rotenone on dopaminergic cell bodies in the SNc, which implies that the primary site of the pathological interaction of mitochondrial deficit with H_2_O_2_ is at the level of dopaminergic terminals in the striatum. Moreover, because the compounds lack direct antioxidant activity (C.L.L. Chai, unpublished observation), it appears that inhibition of self-amplifying pathological cytoplasmic DA efflux and DAQ formation at the striatal nerve terminals could prevent subsequent oxidative damage in the substantia nigra indirectly. This feature could also be beneficial during the early stages of neurodegeneration.

The MPTP model is used widely for preclinical in vivo assessment of anti-parkinsonian effects, due to its high reproducibility and disease-related pathological features [35, 51–53]. MPTP is first taken up by astrocytes and converted there to the toxic metabolite 1-methyl-4-phenylpyridinium (MPP^+^) by the MAO enzyme. MPP^+^ is then released and selectively taken up to the dopaminergic nerve terminals by the dopamine transporters and causes the oxidative damage of dopaminergic neurons [36]. When degeneration of the nigrostriatal dopaminergic pathway was examined in an acute MPTP-induced in vivo PD model in mice, a consistent protective effect was detected by the test compounds, both in terms of dopamine depletion, survival rate and motor function. We have chosen the compound SZV558 for a more detailed characterization and its effect was dose-dependent, with a significant effect detected in lower, 1 mg/kg dose in line with its nanomolar potency to inhibit MAO-B enzyme. Moreover, its protection was more efficient than rasagiline using oral route of application, indicating that its pharmacokinetic properties are suitable for *per os* treatment. Although not all compounds displayed this effect, SZV2220 and SZV2533 had a preferable effect on the level of endogenous dopamine metabolites. In case of rasagiline, the level of DOPAC and HVA remained at a low level after MPTP treatment, which is probably explained by the complete inhibition of MAO-A and MAO-B at this relatively high dose, which prevented the further metabolism of dopamine [54]. In contrast, SZV2220 and SZV2533 restored the level of dopamine metabolites which indicates an enhanced turnover rate and functionality of surviving dopaminergic nerve terminals. As for the behavioral readout, the effect of SZV compounds was comparable to that of rasagiline.

The acute MPTP model used in the previous experiments represents a fast and reliable experimental method to elicit non-apoptotic degeneration of the nigrostriatal dopaminergic pathway affected in PD. However, the progress of the neurodegeneration in human PD patients is initially slow and gradual and take place in both apoptotic and non-apoptotic way. Moreover, because MPTP is converted to the toxic metabolite MPP^+^ by the MAO-B enzyme, the in vivo protective effect of the compounds in the acute model might be simply explained by the inhibition of MPP^+^ formation, but not a genuine neuroprotective action. To clarify this issue, we have analyzed MPTP and MPP^+^ levels in the striatum samples derived from mice subject to pre-treatment with test compounds or saline. As expected, the majority of MPTP has already been converted to MPP^+^ at this time point, and although MPTP was still detectable, it remained at a low level. There was no significant difference in MPP^+^ levels in mice that have been pretreated with SZV2220 or SZV558 18 h before the start of MPTP treatment, when compared to vehicle treated animals. This finding indicates that the compounds had a negligible effect on the MPTP/MPP^+^ ratio under our experimental conditions. Nevertheless we cannot entirely exclude that at an earlier time point after MPTP addition (<72 h), the compounds delayed the formation of MPP^+^ as it is postulated that a substantial part of MPTP is converted to MPP^+^ by MAO-B enzyme [32, 55].

Therefore, two test compounds, SZV558 and SZV2220 were examined in another rodent MPTP model, i.e. in the Tatton-Kish model [32, 33]. The protocol used by this model results in a prolonged, apoptotic degeneration of SNc neurons lasting about 3 weeks after a short course of daily MPTP administration and the putative protective agents are added only after MPTP administration. Consequently, a protective effect clearly independent from the disruption of MPTP uptake and metabolism by MAO-B inhibition could be revealed. Using this protocol, a less severe, but still significant depletion of striatal endogenous dopamine content was found after MPTP treatment in good agreement with previous studies [33]. SZV558 was protective and more efficacious than rasagiline against the depletion of dopamine, even after the administration of MPTP, i.e. when almost all MPTP is already converted to the toxic metabolite MPP^+^ by the MAO-B enzyme, as confirmed by HPLC analyses. This observation indicates that SZV558 has a protective action independent from the inhibition of MAO-B enzyme. To further support this assumption, SZV2220, which is a more potent but less selective inhibitor of MAO-B than SZV558 [24], was less effective in this experimental paradigm.

In the acute and subchronic in vivo MPTP models we have regarded the preservation of the dopamine content by the test compounds as an indicator of increased survival of dopaminergic neurons. To prove their efficacy on dopaminergic cell death in vivo, we have also evaluated the neuroprotective effect of SZV558 against the loss of tyrosine hydroxylase immunoreactivity in the SNc and against PD-like motor symptoms in a chronic MPTPp model. This latest model, differently from acute MPTP treatments that induce a rapid degeneration of dopaminergic nigrostriatal neurons, induces a more progressive degeneration, with neurons continuing to die after completion of toxin administration [27]. Moreover, when MPTP is co-administered with probenecid, which retards the renal and central nervous systems’ clearance of the toxic metabolites of MPTP, the degeneration of dopaminergic neurons takes place over a period of 5–8 weeks. This chronic regimen induces apoptosis, without mortality of mice which survive in a healthy state to the treatment, very important for the subsequent behavioral testing. Moreover, the chronic treatment induces olfactory deficit, one of the symptoms that characterizes the early as well as late stages of PD. Progression in a model with MPTP is a very important requirement since it allows the study of the efficacy of neuroprotective drugs during the progressive dopaminergic neurodegeneration, reproducing human pathology more closely. The chronic model utilized in this study has been well characterized and was validated for utilization in a protocol of drug-induced neuroprotection [30].

The beam-walking test showed that MPTPp increases the number of steps and errors, indicating an impairment of gait. It is interesting to note that the beam-walking test strictly reflects the deficits that characterize PD, such as slowness of movements, indicated by the increased number of steps to traverse the length of the beam, together with an unstable gait, indicated by the number of errors [56]. SZV558, similarly to rasagiline, counteracts the slowness of movement, together with the unstable gait induced by chronic MPTPp. In contrast, no impairment after chronic MPTPp was observed in the grip test, indicating that limb strength was not impaired by the chronic regimen of MPTPp administration and no modifications were produced by either SZV558 or rasagiline. Not surprisingly, no differences were observed in locomotor activity and total motility after chronic MPTPp, as adaptive changes in motility are consistently observed when the DA neuron lesion does not exceed 70–80 % [27, 57]. Moreover, SZV558 counteracted the olfactory deficit induced by chronic MPTPp as measured by the decreasing pellet retrieval time.

The rescue of neurodegeneration was not only indicated by the positive results obtained in the above-mentioned behavioral tests, but also by the immunohistochemical results that showed that dopaminergic neurodegeneration, evaluated through TH immunohistochemistry, was rescued by SZV558. The drug, similarly to rasagiline, completely counteracted the decrease in TH-positive neurons and terminals observed after chronic MPTPp in the SNc and striatum, respectively. Therefore, the results showed a strict association between behavioral and biochemical markers of dopaminergic neurodegeneration. In addition, we used a Nissl stain in order to verify the real death of dopaminergic neurons in the SNc. The results of that analysis confirmed that chronic MPTPp caused a degeneration of dopaminergic cells and that SZV558 produces neuroprotection.

## Conclusions

In conclusion, the novel propargylamines are protective in all in vitro and in vivo models of PD used. Therefore, simultaneous inhibition of MAO-B and oxidative stress-induced pathological dopamine release seems to be a plausible strategy to combat PD (Fig. 8), and to our knowledge this is the first report on compounds specifically interacting with these mechanisms. In addition, neuroprotection by SZV558 is demonstrated in an in vivo chronic model of PD, confirming the therapeutic potential of this compound on neurodegenerative disease.

PD is a multifactorial disease and several risk factors contribute to the vulnerability of DA neurons [58], therefore, SZV558 by affecting multiple targets, may, through these differentiated mechanisms have a more favorable action than other MAO-B inhibitors as neuroprotective agent in the therapy of PD.

## Methods

### Animals

All studies were conducted in compliance with the Directive 2010/63/EU and ARRIVE guidelines and were approved by the local Animal Care Committee of the Institute of Experimental Medicine (Budapest, Hungary, Permission No: 22.1/3671/003/2008) or by the Ethical Committee of the University of Cagliari. Animals were kept under standard laboratory conditions (12 h light/12 h dark cycle) with food and water *ad libitum*. All efforts were made to minimize animals suffering and reduce the number of animals used.

### [^3^H]dopamine release experiments in rat striatal slices

The [^3^H]dopamine ([^3^H]DA) release experiments were performed as described in our previous studies [18, 19]: male Wistar rats (180–220 g, obtained from the local animal house) were decapitated under light CO_2_ anesthesia and the brain quickly removed into ice-cold Krebs’ solution. The striatum was dissected and sliced into 400 μm sections with a McIlwain tissue chopper. Striatum slices then were pretreated with rotenone (10 μM, Sigma) for 60 min, and subsequently incubated in 1 ml of Krebs’ solution containing 5 μCi [^3^H]DA (spec. activity: 60 Ci/mmol, ARC, Saint Louis, MO, USA) for 45 min continuously gassed with a mixture of 95 % O_2_ and 5 % CO_2_ at 37 °C. After incubation, the slices were transferred to tissue chambers and perfused continuously with modified Krebs solution at a rate of 0.5 ml/min. After a 60-min pre-perfusion, 3-min samples were collected and assayed for [^3^H]DA. Oxidative stress was modelled by addition of H_2_O_2_ (250 μM) to the Krebs solution, starting at the 9^th^ min of collection. Compounds were added to the perfusion fluid 18 min prior to H_2_O_2_ application. In other experiments, slices were subjected to electrical field stimulation (EFS1, EFS2) twice, using a Grass S88 stimulator, with the following parameters: 25 V, 1 msec, 2 Hz, 240 shocks and SZV558 was administered 18 min before EFS2 and onwards. Tritium was measured with a Packard 1900 TR liquid scintillation counter using an internal standard.

The release of tritium was calculated in Bq/g and expressed as percentage of the amount of radioactivity in the tissue at the time of sample collection (fractional release, FR %). The evoked release of [^3^H]DA and its tritiated metabolites in the presence/absence of drugs was expressed as the net release evoked by H_2_O_2/_EFS and was calculated by the area-under-the-curve (AUC) method, i.e. subtracting the resting release calculated in the prestimulation period, from the release measured during H_2_O_2_/EFS.

### HPLC analysis

DA and DA metabolites in the tissue extract and superfusate were measured using electrochemical and liquid scintillation detection. Tissue slices were immediately frozen in liquid nitrogen after termination of the experiment. Weighed frozen tissue was homogenized in an appropriate volume of ice-cold 0.1 M PCA that contained theophylline (as an internal standard) at 10 nmol/ml concentration and 0.5 mM sodium metabisulphite (antioxidant for biogenic amines). The suspension was centrifuged at 300 g for 10 min at 0–4 °C. The perchloric anion was precipitated by addition of 10 μl of 1 M KOH to 190 μl supernatant. Precipitate was then removed by centrifugation. Supernatant was kept at −20 °C until analysis. The pellet was saved for protein measurement according to [59].

During the collection time the tissue perfusate was acidified with 10 μl of 1 M perchloric acid. The perfusion fluid was centrifuged at 300 g for 10 min at 0–4 °C; the supernatant was kept at −20 °C until analysis. The identification of tritium labeled compounds was based upon known retention times of unlabeled standards.

For the analysis, 1050-μl sample volumes were diluted with 50 μl of 10^−5^mol/l theophylline as an internal standard and 1000 μl injected. The analytic separations were performed on a Supelcosil LC-C18DBC18 DB (150 × 4.6 mm I.D., 3 μm particle size) column from Sigma-Supelco (Steinheim, Germany) as described previously [20]. The detector layouts were connected to Gilson 715 and Agilent data acquisition systems. Radioactivity of each 1-min effluent samples was determined by liquid scintillation counting. The [^3^H]activity of superfusate samples were quantified using the following formula:

$$ \mathrm{R} = 100 \times \left({\mathrm{sum}\ \mathrm{A}}_{\mathrm{i}}\right)/\ \left({\mathrm{A}}_{\mathrm{o}}-{\mathrm{A}}_{\mathrm{i}\mathrm{nj}}\right), $$

Where the activity of sample is (A_o_); the activity of the injection waste is (A_inj_); the activity of 1 min effluent is (A_i_). Content of DA, 3,4-dihydroxyphenylacetaldehyde (DOPAL), 3,4-dihydroxyphenylethanol (DOPET), 3,4-dihydroxyphenylacetic acid (DOPAC), homovanilic acid (HVA), DAQ, tyramine (TYR), 3-methoxityramine (3-MT) and beta-phenyl-ethylamine (PEA) were expressed in pmol/mg protein or as percentage of the total tritium label measured in the sample.

MPTP and MPP^+^ levels in tissue slices was determined by HPLC with UV detection, and the UV detector signal (set at 253 nm) was used for the quantifications. The standards MPTP-HCl and MPP^+^ iodide were purchased from Sigma-Aldrich. Data were calculated by internal standard calibration, and expressed as pmol/mg protein.

### Tyrosine hydroxylase (TH) immunohistochemistry following in vitro incubation

Male Wistar rats (200–220 g) were used in the experiments. The animals were decapitated under light CO_2_ anesthesia and brains were quickly removed and incised in the middle, so the hemispheres could be handled separately. Blocks containing the substantia nigra were dissected. There were five treatment groups: control, incubation with rotenone, rotenone + H_2_O_2_, rotenone + H_2_O_2_ + rasagiline (100 nM) and rotenone + H_2_O_2_ + SZV558 (100 nM). Control tissue was incubated in Krebs solution (60 min, 37 °C), then perfused with the Krebs solution for 120 min. The rotenone group was treated with 10 μM rotenone during the incubation period (60 min, 37 °C), then the same protocol was continued as for the control group. The rotenone + H_2_O_2_ treated group was identical to the rotenone protocol, except that perfusion with Krebs solution (70 min) was followed by perfusion with Krebs solution containing 250 μM H_2_O_2_ (50 min). In cases of rotenone + H_2_O_2_ + rasagiline and rotenone + H_2_O_2_ + SZV558 groups, after incubation with rotenone, the tissues were perfused with Krebs solution (50 min), then were subjected to 100 nM rasagiline/SZV558 perfusion (20 min), and then to 250 μM H_2_O_2_ and 100 nM rasagiline/SZV558 perfusion (50 min). Each incubated block was immersion fixed with 4 % paraformaldehyde (PFA) overnight, then washed with 0.1 M phosphate buffer (PB). The whole substantia nigra was sectioned with a vibratome at 40 μm thickness between and all coronal midbrain sections were collected in a systematic random fashion. Ten consecutive sections were used for immunostaining. All sections were processed together and the same batch of reagents was used. Non-specific endogen peroxidase activity was blocked by 0.3 % H_2_O_2_ in methanol for 20 min. To reduce non-specific binding Vector blocking solution (2.5 % normal horse serum) was applied for 2 h at room temperature. The primary antibody, anti-tyrosine hydroxylase (rabbit polyclonal IgG, Merck Millipore) was used in a 1:1000 dilution in 0.1 M PB overnight at 4 °C. After carefully washing three times with 0.1 M PB, the ready-to-use secondary antibody (The ImmPRESS Universal Antibody Kit, anti-mouse/rabbit) and ImmPACT DAB as chromogen (both purchased from Vector Laboratories, Burlingame, CA) was applied according to the manufacturer’s instructions. The sections were dried on glass slides, cleared with xylene and coverslipped with Depex (Sigma, Aldrich Co, St. Louis, MO, USA).

Digitalization was performed by means of a Pannoramic P250 scanner (3DHISTECH, Budapest, Hungary) using 9 optical layer with a resolution of 0.11 μm/pixel. The same areas and number of sections were investigated. Pictures were taken at 40x magnifications. The contours of *substantia nigra pars compacta* were traced in every other serial section (*n* = 5, spaced at 80 μm from each other) according to the online-available Paxinos Rat Brain Atlas/https://gaidi.ca/rat-brain-atlas/?ml=2&ap=−5.4&dv=8.7/. The stained cells were counted manually by two independent investigators, marked with a marker counter function of the Pannoramic Viewer 1.15.4 digital slide viewer software application provided by 3DHISTECH, Budapest, Hungary.

### In vivo acute 1-methyl-4-phenyl-1,2,3,6-tetrahydropyridine (MPTP) model

In this set of experiments, a widely used acute MPTP protocol was applied, established by numerous previous studies [32]. Adult (2–3 month old, 30 g) male C57/Bl6 mice (Charles-River, Hungary) were used and randomly assigned to pre-defined experimental groups. Briefly, MPTP (Sigma) was injected (4x20 mg/kg i.p) 2 h apart, and 72 h after the last MPTP treatment animals were euthanized under light CO_2_ anesthesia and biogenic amine content of the striatum analyzed by HPLC-EC analysis. Control animals received saline injections in an identical way. The motor function was evaluated 2 h and 1 day after the last MPTP treatment by the open field and rotarod tests, respectively. Test compounds were applied in a single, 0.1-20 mg/kg dose i.p. 18 h prior to the MPTP treatment, or *per os* (20 mg/kg) using a gavage. In other experiments test compounds were applied in a single dose (10 mg/kg i.p.) 2 h, or 42 h before the first dose of MPTP. In all experiments animals were daily monitored for general health and overt side effects.

### Behavioral analyses

#### Open field test

The open field test is the most commonly used method to measure alterations of behavioral activity after MPTP treatment [60]. Animals were transferred to the experimental room at least 3 days prior to testing. C57/Bl6 mice were treated with MPTP as described above and placed 2 h after the final MPTP injection for 30 min into the open field arena, following a protocol used to test putative antiparkinsonian drugs on locomotion [61, 62]. Experiments were performed in the light phase under dimmed lights (~3 lux). Each animal was placed in the center of a nontransparent plexiglas arena (dimensions: 40x40x40 cm) for a habituation period of 30 min, and the locomotor activity of animals recorded for 30 min using a video camera positioned above the arena. The total distance in meters was provided for the 30 min of the experiment.

#### Rotarod test

Motor coordination was tested on the IITC (Woodland Hills, CA, USA) Rotarod Apparatus, which enables the simultaneous examination of five mice or rats. Because mice with striatal dopamine depletion show only mild or no deficit on the typical accelerating rotarod test, the modified protocol of Shiotsuki [63] was followed. In this version mice are tested on a larger drum with fixed speed to obtain a steep learning curve and therefore this test is more selective for motor skill learning rather than maximal gait performance. The apparatus consists of five separated compartments, with an 8 cm diameter rotating rod 25 cm above the base of the apparatus. Motor coordination of animals was tested for 180 s with a fix speed of 10 rpm. Mice were acclimatized to the rotarod in two trials (180 s) per days for 2 consecutive days before the start of the experiment. On the test day, 1 h before drug administration, baseline latencies to fall were determined. The animals were then treated with sterile saline or test compounds, followed by MPTP as described above. 6 and 24 h after the final MPTP treatment, the falling latency was measured again in the 180 sec test period. The latency time to fall was expressed in seconds.

#### In vivo subchronic MPTP model

Two to three month old, male C57/Bl6 mice (Charles-River, Hungary) were treated daily with saline or MPTP (30 mg/kg i.p) for 5 consecutive days before the test compounds were delivered for 21 days [33, 34]. The MPTP group received vehicle treatment for the same period after the MPTP treatment. In contrast to the acute MPTP model, using this protocol, dopaminergic neurons degenerate in a delayed, apoptotic manner [34]. After the experiments, endogenous dopamine content in the striatum was determined using the HPLC method described above.

#### Chronic protocol of MPTP plus probenecid (MPTPp)

Experiments were performed on 3-month-old male C57Bl/6 J mice (Charles River, Italy) treated with vehicle plus probenecid, or MPTP (25 mg/kg i.p.) plus probenecid (100 mg/kg i.p.), administered 30 min before each MPTP administration (MPTPp), twice a week for 5 weeks, alone, or in the presence of the SZV558 (1 mg/kg i.p.) or rasagiline (1 mg/kg i.p.) administered 18 h before each MPTPp administration. At the end of treatment, mice were tested behaviorally (motility test, beam-walking test, inverted grid test and olfactory test) to evaluate the motor and olfactory performance impairments. Animals were sacrificed 3 days after the last administration of MPTPp. Dopaminergic neurodegeneration was studied in the striatum and SNc by immunohistochemical evaluation of TH-positive neurons and Nissl staining.

### Spontaneous motor activity: motility test

Spontaneous motility was assessed 2 days before the MPTPp treatment and 1 day after the last MPTPp administration, in a quiet isolated room. Mice were placed individually in plexiglass cages (length 47 cm, height 19 cm, width 27 cm), with a metal grid over the floor, and equipped with infrared photocell emitters-detectors situated along the long axis of each cage (Opto-Varimex Mini; Columbus Instruments). The interruption of a photocell beam was detected by a counter that recorded the total number of photocell beam interruptions. The counter recorded two different types of motor activity: locomotor activity due to the locomotion of the mouse along the axes of the cage and total motor activity due to locomotion plus non-finalized movements (stereotyped behaviors, such as grooming, rearing, and sniffing). The counter recognized the stereotyped movements because of the continuous interruption of the same photocell beam, whereas locomotion along the cage produced interruptions of different photocell beams. Motility was detected as soon as the mouse entered into the cage and was evaluated for 60 min [64].

### Beam-walking test

The motor performance and coordination of mice were evaluated with the beam-walking test [65]. In this test, mice were trained to traverse the length of a plexiglass beam that was divided into four sections of 25 cm each (1 m total length). Each section of the beam had a different width: 4, 3, 2, and 1 cm; the beam was placed on a table and ended in the animal’s home cage. Mice received 2 days of training before testing. On the first day, mice received two assisted trials, involving the placement of the mouse on one extremity of the beam with the home cage in close proximity to the animal. This encourages forward movement along the beam. After two assisted trials, mice were able to traverse the entire length of the beam unassisted. Days 1 and 2 of training ended when all animals had completed five unassisted runs across the entire length of the beam. To increase the difficulty further, on the day of the test, a mesh grid (1 cm squares) of corresponding width was placed over the beam surface. The test was performed 2 days after the last MPTPp administration and mice were videotaped for a total of five trials. An error was counted when, during a forward movement, a limb slipped through the grid. By scoring each limb slip individually, the severity of the error could be measured. The number of steps and the number of errors were calculated across all five trials and averaged for each group [65].

### Inverted grid test to evaluate grasp strength

The inverted grid test was used to assess skilled forepaw use, especially related to the distal musculature and digit manipulations. Mice were placed in the center of a horizontal square grid (15 cm^2^) consisting of a wire mesh (mesh 0.5 cm^2^) surrounded by wooden walls. The grid was placed 20 cm above a tabletop and was rotated upside down allowing mice to move freely. The time the mice took before falling down was recorded. If a mouse fell from the mesh grid within 10 s, additional trials were allowed (maximum: three trials) within an interval of 5 min; in this case, latencies before falling were measured. The mean ± SEM of three trials was calculated. Moreover, for each trial, the number of steps and forelimb fault per step was rated and compared with controls. No training was performed, but a pre-test was carried out [66]. The time the mice took before falling down was measured 2 days after the last MPTPp administration.

### Olfactory test

Mice were food-deprived for 20 h before the olfactory test. The test was conducted in a clean plastic cage (length 42 cm, height 15 cm, width 24 cm). A smelling pellet was buried under the bedding (1 cm) in a cage corner. The mouse was positioned in the center of the cage and the time to retrieve the pellet and bite it was measured [29]. The retrieval time of the buried pellet was measured 3 days after the last MPTPp administration.

### Immunohistochemistry and cresyl violet for Nissl staining

Three days after the last administration of MPTPp, the mice were anesthetized with chloral hydrate (400 mg/kg i.p.), transcardially perfused with 4 % PFA in PB (0.1 M, pH 7.4), and their brains removed and used for immunohistochemistry. Coronal sections (40 μm thick) were cut on a vibratome. Free-floating sections were incubated overnight with TH antibody (polyclonal rabbit anti-TH, 1:1000, Millipore, USA).

The primary antibody was prepared in PBS plus Triton solution containing normal goat serum. After careful washing, the sections were incubated in proper biotinylated secondary antibody (Vector, UK). For visualization, avidin-peroxidase protocol (ABC, Vector, UK) was applied, using 3,3′-diaminobenzidine (Sigma, Italy) as chromogen. After washing, the sections were mounted on gelatin-coated slides, air-dried, dehydrated in ascending concentrations of ethanol, and cleared with xylene [67].

Adjacent SNc sections were stained with cresyl violet for the Nissl staining to evaluate cell death in this area. For TH and cresyl-violet-stained cells immunohistochemistry in the SNc, three sections were sampled (anterior–posterior: −2.92 to −3.28 mm from bregma) according to the atlas of Paxinos and Franklin 2001. For each mouse, three sections from the striatum (anterior-posterior: 1.10 mm to 0.62 mm from bregma) were analyzed for TH.

### Analysis of TH-positive fibers in the striatum

Images were digitized in gray scale with a video camera (Pixelink PL-A686) and TH immunoreactivity analysis was performed using the Scion Image analysis program (Scion Corp., USA). The average gray values from white matter were subtracted from each section to correct for background immunoreactivity. For each level of striatum, the obtained value was first normalized with respect to vehicle, and values from different levels were averaged thereafter.

### Analysis of TH-positive cells and Nissl staining in the SNc

Three sections of SNc were captured at 10× magnification; in each section, the whole left and right SNc area were analyzed. Images were digitized (PL-A686 video camera, Pixelink, Canada) under constant-light conditions. The stained cells were counted manually by a blind experimenter. The number of TH-positive cells and Nissl stained neurons was obtained separately for each SNc level. Thereafter, in order to obtain an average value from all levels analyzed, the number of cells/level from each mouse was normalized with respect to the vehicle. Values from the three levels were then averaged to generate a mean.

#### Statistical analysis

Data are expressed as mean ± SEM with *n* = number of identical experiments. Statistical analyses were performed using Prism-3 software (version 3.00, Graph Pad, San Diego, CA). Student’s *t* test (pairwise comparisons) and one-way analysis of variance (ANOVA, multiple comparisons) were used as a statistical analysis, as appropriate. Behavioral results were statistically compared with a one-way analysis of variance (ANOVA), followed by Newman-Keuls post hoc test, for comparison between experimental groups. For neurochemical analyses and immunohistochemistry experiments, results were statistically analyzed by one-way ANOVA followed by Dunnett/Tukey’s post hoc test (for unequal N). Results were considered significant at *P* < 0.05.

#### Materials

The following drugs were used:

Test compounds, i.e. SZV2220, SZV558, SZV2358, SZV2419, SZV2435 and SZV2533, (for structural formulae, see Table 1) and rasagiline were synthesized according to that described earlier [24]. Rotenone, MPTP and MPP^+^ were obtained from Sigma-Aldrich (St. Louis, MO, USA). Probenecid was obtained from Sigma and dissolved in 5 % NaHCO_3_. H_2_O_2_ and salts used in the Krebs solution were obtained from Reanal ZRT., Budapest, Hungary. The composition of the modified Krebs solution was as follows: NaCl 113, KCl 4.7, CaCl_2_ 2.5, KH_2_PO_4_ 1.2, MgSO_4_ 1.2, NaHCO_3_ 25, Na_2_EDTA 0.03, ascorbic acid 0.3, and glucose 11.5 mM. All solutions were freshly prepared on the day of use.

## Additional file

Additional file 1: Table S1. The duration of action of SZV558 (10 mg/kg) i.p. and the effect of its metabolites on endogenous dopamine content in the striatum and on the survival of animals after in vivo MPTP treatment (DOC 31 kb)

## Abbreviations

ANOVA: Analysis of variance
AUC: Area-under-the-curve
PEA: Beta-phenyl-ethylamine
CTRL: Control
COMT: Catechol-o-methyltransferase
DOPAL: 3,4-dihydroxyphenylacetaldehyde
DOPET: 3,4-dihydroxyphenylethanol
DOPAC: 3,4-dihydroxyphenylacetic acid
DA: Dopamine
DAQ: Dopamine quinone
EFS: Electrical field stimulation
[^3^H]DA: [^3^H]dopamine
HVA: Homovanilic acid
L-DOPA: levo-DOPA
3-MT: 3-methoxityramine
MAO: Monoaminooxidase
MAO-A: Monoaminooxidase–A
MAO-B: Monoaminooxidase-B
MPP^+^: 1-methyl-4-phenylpyridinium
MPTP: 1-methyl-4-phenyl-1,2,3,6-tetrahydropyridine
MPTPp: 1-methyl-4-phenyl-1,2,3,6-tetrahydropyridine plus probenecid
6-OHDA: 6-hydroxidopamine
PD: Parkinson’s disease
PFA: Paraformaldehyde
PB: Phosphate buffer
PBS: Phosphate buffer solution
ROS: Reactive oxygen species
ROT: Rotenone
TH: Tyrosine hydroxylase
TYR: Tyramine
