# Supplementary material for: Novel (Hetero)arylalkenyl propargylamine compounds are protective in toxin-induced models of Parkinson’s disease
Source: Mol Neurodegener. 2016 Jan 13;11:6. doi: 10.1186/s13024-015-0067-y (PMC4711075; doi:10.1186/s13024-015-0067-y)
Supplement: Additional file 1: Table S1. — The duration of action of SZV558 (10 mg/kg) i.p. and the effect of its metabolites on endogenous dopamine content in the striatum and on the survival of animals after in vivo MPTP treatment (DOC 31 kb) [file 13024_2015_67_MOESM1_ESM.doc]

**Table S1.** The duration of action of SZV558 (10 mg/kg) i.p. and the effect of its metabolites on endogenous dopamine content in the striatum and on the survival of animals after *in vivo* MPTP treatment.

In these experiments, MPTP was injected 4 times, 2 h apart (20 mg/kg i.p.) in adult C57/Bl6 mice and the survival of the animals and biogenic amine content of the striatum analyzed 72h after the final injection by HPLC analysis, as described in the Materials and Methods. Test compounds were applied in a single dose (10 mg/kg i.p.) 2 h, 18 h or 42 h before the first dose of MPTP, as indicated.

| **Treatment** | **DA (pmol/mg protein)** | **Survival (%)** |
| --- | --- | --- |
| Saline | 602.05±31.86 | 100% |
| MPTP | 88.56±24.89^+++^ | 50% |
| SZV558 2 h | 205.76±13.98 | 100% |
| SZV558 18 h | 385.63±35.99^***^ | 100% |
| SZV558 42 h | 183.82±35.53 | 100% |
| rasagiline 18 h | 319.25±67.05^***^ | 100% |
| rasagiline 42 h | 114.79±4.20 | 100% |
| SZV557 18 h | 176.76±18.39 | 100% |
| SZV1680 18 h | 18.10±1.82 | 55% |

Dopamine content is expressed as pmol/mg protein. Symbols represent significant changes from saline treated (^+++^P<0.001), and MPTP treated (^***^P<0.001) treated animals, respectively. Statistical analysis: one-way ANOVA followed by the Tukey test. Number of independent experiments: 5-8/group.
